# DNA damage, oxidative stress, and inflammation in children with celiac disease

**DOI:** 10.1590/1678-4685-GMB-2018-0390

**Published:** 2020-06-10

**Authors:** Sharbel Weidner Maluf, Danilo Wilhelm, Eduardo Benedetti Parisotto, Guilherme da Silva de Medeiros, Carolina Hilgert Jacobsen Pereira, Flora Troina Maraslis, Carlos C. Dornelles Schoeller, Julia Savan da Rosa, Tânia Silvia Fröde

**Affiliations:** 1 Universidade Federal de Santa Catarina, Hospital Universitário, Laboratório de Genética, Florianópolis, SC, Brazil; 2 Universidade Federal de Santa Catarina, Departamento de Ecologia e Zoologia, Florianópolis, SC, Brazil; 3 Universidade Federal de Mato Grosso do Sul, Faculdade de Ciências Farmacêuticas, Alimentos e Nutrição, Campo Grande, MS, Brazil; 4 Hospital Infantil Joana de Gusmão, Florianópolis, SC, Brazil; 5 Universidade Federal de Santa Catarina, Centro de Ciências de Saúde, Departamento de Análises Clínicas, Florianópolis, SC, Brazil

**Keywords:** Celiac disease, biochemical markers, genotoxicity, reactive oxygen species (ROS), inflammatory markers

## Abstract

The objective of this study was to evaluate the level of genomic instability in patients with celiac disease and to establish a relationship between inflammation, oxidative stress, and DNA damage in these patients. Myeloperoxidase (MPO) activity, adenosine deaminase, nitric oxide (NOx), thiobarbituric acid, catalase (CAT), superoxide dismutase (SOD), glutathione peroxidase (GPx), and DNA damage were evaluated in peripheral blood samples from 47 celiac disease patients and 31 controls. Patients with celiac disease presented higher levels of DNA damage in comparison to controls (*p*=0.023). This difference was also observed for markers of oxidative stress, such as CAT (*p*=0.011) and SOD (*p*=0.013), and inflammatory markers such as MPO (*p* < 0.001) and NOx (*p*=0.009). Positive correlations were found between DNA damage levels and the values of CAT (r=0.405; *p*=0.009) and SOD (r=0.516; *p* < 0.001). Positive correlations were also found between GPx and NOx (r=0.349; *p*=0.030) and MPO and NOx (r=0.239; *p*=0.039). CAT and NOx showed a negative correlation (r= −0.315; *p*=0.042). In conclusion, intestinal inflammation can have systemic effects, causing an imbalance between oxidant and antioxidant markers, which may promote increased levels of DNA damage.

## Introduction

Celiac disease (CD) is a chronic inflammatory condition of the small intestine caused by a reaction to gluten exposure. There are many genetic and environmental factors that can interact to cause the disease (Sollid and Jabri, 2005; Van Heel and West, 2006). Histologically, the affected small intestine presents with villous atrophy, crypt hypertrophy, flat mucosa, and intraepithelial lymphocytosis, which leads to malabsorption of micro- and macronutrients (Bottaro *et al.*, 1999; Murray, 1999). The treatment for CD is a gluten-free diet (GFD), which, in most cases, leads to the disappearance of symptoms. A GFD also prevents other autoimmune diseases, osteoporosis, and gastrointestinal cancer, which are CD-related conditions that may occur (Wright, 1995; Ryan and Kelleher, 2000).

Some gliadin peptides possess the ability to penetrate cells by endocytosis (Schumann *et al.*, 2008; Heyman and Menard, 2009). Peptide accumulation in lysosomes leads to increased levels of reactive oxygen species (ROS) (Zimmer *et al.*, 2010). In addition to ROS, reactive nitrogen species (RNS) are also referred to as oxidants due to their ability to remove electrons from biological molecules, thus promoting a nitrosative stress (Halliwell and Gutteridge, 2007). When oxidizing compounds are high in relation to antioxidant defenses oxidative stress occurs, either due to increased ROS and RNS or decreased antioxidant defenses. Antioxidant defenses can come from endogenous sources such as catalase (CAT), superoxide dismutase (SOD), glutathione peroxidase (GPx), glutathione reductase (GSR), and reduced glutathione (GSH), and dietary sources, such as vitamins, minerals, and polyphenols (Halliwell and Gutteridge, 2007; Kerasioti *et al.*, 2017). SOD catalyzes the conversion of the superoxide anion (O_2_^•-^) into hydrogen peroxide (H_2_O_2_), while CAT reduces H_2_O_2_ to water (H_2_O) and oxygen (O_2_). GPxs, in turn, are able to reduce H_2_O_2_ and lipid hydroperoxides by oxidizing GSH into oxidized glutathione, and subsequently, GSR can regenerate GSH (Halliwell and Gutteridge, 2007; Kerasioti *et al.*, 2017).

Studies have shown that oxidative stress is one of the mechanisms that can play a role in gliadin toxicity. Gliadin, a class of proteins present in wheat, can trigger oxidative stress and induce the release of pro-inflammatory cytokines (Shan *et al.*, 2002; Ciccocioppo *et al.*, 2005). ROS can lead to a subsequent DNA damage (Cooke *et al.*, 2006). Chronic inflammation can be linked to carcinogenesis and it has been reported that it may be involved in the development of about one third of all cancer cases (Ames *et al.*, 1993; Coussens and Werb, 2002). In this regard, patients with celiac disease have an increased risk of cancer (Han *et al.*, 2015).

There is evidence that DNA damage may be used as a predictive marker of cancer development (Olinski *et al.*, 2003). Single cell gel electrophoresis (SCGE), also known as the comet assay, provides a reliable method for assessing DNA damage quantitatively in single cells. It requires viable but not growing interphase cells, and can be applied to terminally differentiated cells (Ross *et al.*, 1995). Lymphocytes are excellent cells to analyze markers of DNA damage exposure because they circulate for years or even decades through different organs and accumulate DNA damage during their lifespan (Natarajan and Obe, 1980; Carrano and Natarajan, 1988). Much evidence has been generated about the association of micronucleus induction (Murgia *et al.*, 2008) and DNA damage detected by the comet assay (Gunasekarana *et al.*, 2015) with the development of cancer.

The overall objective of this study was to evaluate biochemical markers of inflammation, nitro-oxidative stress, and levels of DNA damage in peripheral blood of patients with celiac disease. Several studies have demonstrated the increase of oxidative stress in celiac disease (Ciccocioppo *et al.*, 2005; Kerasioti *et al.*, 2017). Here, we assessed the correlation of inflammatory markers with increased levels of DNA damage, evidenced through the comet assay.

## Subjects and Methods

### Subjects

**Figure 1:**
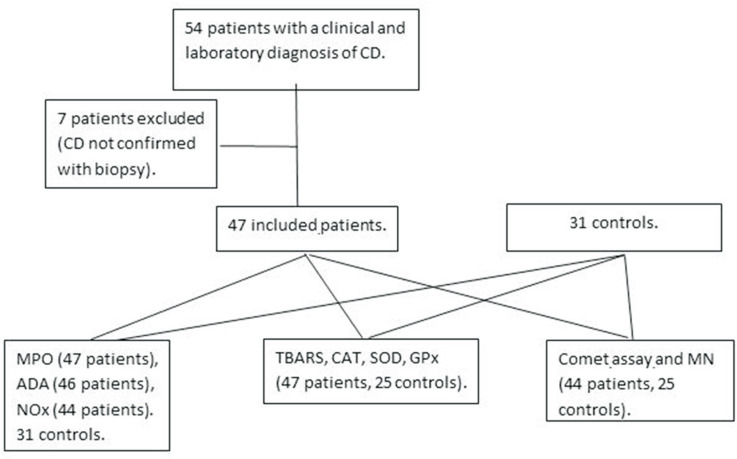
Study flow chart.

Blood samples from 47 patients with CD who attended the Hospital Infantil Joana de Gusmão (HIJG), Florianopolis in Southern Brazil, were collected. The patient group consisted of 18 boys and 29 girls, with a mean age of 7.12 ± 2.76 years. The control group consisted of 12 boys and 19 girls, with a mean age of 6.42 ± 2.33 years (Figure 1). For the patient group, the samples were collected at the moment of first endoscopy with biopsy for diagnosis of CD and the patients were included in the study after the confirmation of the diagnosis. No child was being treated with medications and none was obese or overweight. The control group was composed by healthy children who were referred for routine examinations and whose parents agreed to participate in this study. This work represents a prospective, case-control study. This study was approved by the Research Ethic Committee of the Universidade Federal de Santa Catarina, SC, Brazil. A written informed consent form was read and signed by the legal guardians of all study participants, who received a copy of the consent form with general information about the study signed by the principal investigator.

Three tubes (4 mL) of blood were collected from each individual: one with heparin (protected from light) for the analysis of DNA damage, one with EDTA for analysis of oxidative stress, and one without anticoagulant (with separating gel), for the analysis of inflammatory markers. All samples were immediately centrifuged after collection and serum was placed in liquid nitrogen for further analysis.

### Quantification of myeloperoxidase activity (MPO)

MPO activity in blood samples was measured according to the method developed by Rao *et al.* (1993). Briefly, standard samples with different concentrations of myeloperoxidase (from human neutrophils, Sigma: M6908) were prepared in order to obtain a standard curve in the range of 0.07–140 mlU/mL. Serum aliquots (40 μL) and standards were transferred to cuvettes and the reaction was initiated with the addition of 360 μL of assay buffer (0.167 mg/mL of o-dianisidine 2HCL and 0.0005% H_2_O_2_). The reaction was stopped with 1% sodium azide. Subsequently, the samples were centrifuged at 50 *g* for 5 min, the supernatants were separated, and absorbance change rates (450 nm) were determined on an ELISA plate reader (Saleh *et al.*, 1996). Myeloperoxidase activity was estimated by interpolation from the standard curve described above. Results are reported as mIUmL^−1^.

### Quantification of adenosine-deaminase activity (ADA)

Initially, standard samples (final volume of 500 μL) with different volume concentrations of NaH_2_PO_4_H_2_O (35 mM), Na_2_HPO_4_H_2_O (15 mM), and NH_3_SO_4_ (15 mM) were prepared to obtain a standard curve in the range of 10–50 U/L. Serum samples (20 μL) were transferred to cuvettes, and a reaction was initiated by the addition of adenosine phosphate buffered solution (pH 6.5, 500 μL, composition: NaH_2_PO_4_H_2_O (35 mM), Na_2_HPO_4_12H_2_O (15 mM), and adenosine (0.5 mM). After incubation for 1 h at 37 °C, the reaction was stopped by the addition of 1000 μL of phenol solution (1 mM), nitroprussiate (0.17 mM), and alkaline buffer (1 mL NaOCl, 11 mM). This solution (final volume 2 mL) was also added to the cuvettes with the different standard samples. Afterward, the absorbance change at 620 nm was determined. ADA activity was estimated by interpolation from the standard curve by colorimetric measurements in an ELISA plate reader (Organon Teknika, Roseland, NJ, USA) (Giusti and Galanti, 1984). Results are reported in U/L.

### Quantification of nitrate/nitrite levels (NOx)

Quantification of the nitric oxide products in the serum was carried out according to the methodology described by Green *et al.* (1982). Fluid samples (0.1 mL) were transferred to cuvettes, and 0.05 M vanadium chloride (0.15 mL) in 1.0 M HCl (0.05 mL) was added to reduce nitrate to nitrite. Immediately, Griess reagent (150 μL of 0.004 M naphthyl ethyl endiamide dihydrochloride in H_2_O and 0.06 M sulphanilamide (150 μL) in 0.03 M H_3_PO_4_, vol. 1:1) were added. After incubation at 37 °C for 45 min, the reaction was transferred to a microplate. Nitrite concentrations were estimated by interpolation from a standard curve of sodium nitrite (0-150 μM) by colorimetric measurements at 540 nm in an ELISA plate reader (Organon Tecknika, Roseland, New Jersey, USA). Results are reported as μM.

### Quantification of lipoperoxidation levels (TBARS)

The endogenous lipid oxidation was evaluated in the plasma by determination of thiobarbituric acid-reactive substances (TBARS) according to the method described by Bird and Draper (1984). In this protocol, products of lipid peroxidation react with thiobarbituric acid producing a pink Schiff base that was measured spectrophotometrically at 535 nm, in an UV/Visible spectrophotometer GBC-916 (GBC Scientific Equipment, Dandenong, Australia). Concentrations were expressed as mmol of TBARS/mL.

### Quantification of antioxidant enzymes

#### Catalase activity

The catalase (CAT) activity was determined in whole blood according to the methodology described by Aebi (1984). The reaction is based on the breakdown of hydrogen peroxide (dissolved on phosphate buffer: mmol: NaCl 137, KCl 2.7 and phosphate buffer salts 10) in the presence of the enzyme (CAT) in the blood resulting in the decrease of a freshly prepared H_2_O_2_ solution (10 mM). CAT activity was measured as the change in optical density for 3 min at 240 nm in a UV/Visible spectrophotometer GBC-916 (GBC Scientific Equipment, Dandenong, Australia). Enzyme activity was expressed as mmolmin^−1^mL^−1^.

#### Superoxide dismutase activity

Superoxide dismutase (SOD) activity was analyzed in whole blood in accordance with the method described by Misra and Fridovich (1972) and modified by Boveris *et al.* (1983). This reaction is based on the epinephrine oxidation (pH 2.0 to pH 10.0) produced by superoxide anion from the xanthine/xanthine oxidase system that generates a pink chromophore named formazan. In this protocol, the epinephrine-adrenochrome transition is inhibited by superoxide dismutase present in the blood. Absorbance was monitored for 3 min at 480 nm in a UV/Visible spectrophotometer. The arbitrary unit of the enzyme activity was expressed as 50% of auto-oxidation inhibition of epinephrine-adhenochrome formation.

#### Glutathione peroxidase activity

Glutathione peroxidase (GPx) activity was analyzed in whole blood using the method described by Flohe and Gunzler (1984). Briefly, this method is based on the tertbutyl hydroperoxide (t-BuOOH) dismutation via oxidation of reduced glutathione (GSH) to oxidized glutathione (GSSG). This oxidation is further catalyzed by GPx resulting in the decrease of absorbance that was evaluated at 340 nm in a UV/Visible spectrophotometer. In this protocol, to avoid over-evaluation of the enzyme activity due to the hemoglobin oxidation present in the blood, 50 mM of KCN was added to the cuvette. The unit of enzyme activity was μmolmin^−1^mL^−1^.

### Comet assay

**Figure 2:**
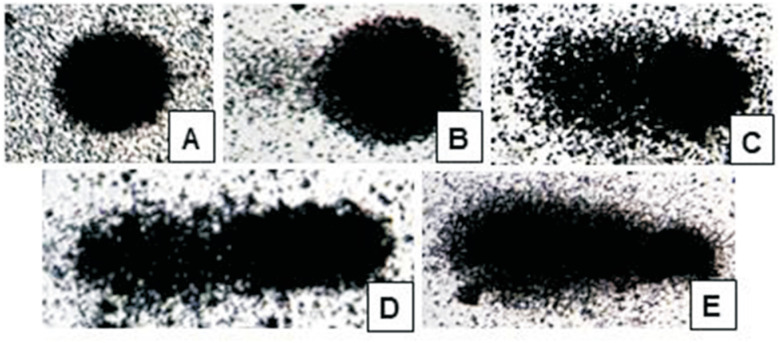
Comet assay: nuclei of leucocytes exhibiting different levels of DNA damage. A: score 0 (no migration); B: score 1; C: score 2; D: score 3, and E: score 4 (maximal migration).

The comet assay protocol was described by Singh *et al.* (1988). The assay was carried out as described for *in vivo* samples (Tice *et al.*, 2000; Hartmann *et al.*, 2003). Blood samples were mixed with low-melting point agarose, spread onto agarose-precoated slides, gently covered with cover slips and placed in a cold tray. Once the samples had solidified, the cover slips were removed and the slides left to stand in lysis buffer (2.5 M NaCl, 100 mM EDTA, 10 mM Tris, pH 10.2, to which 1% Triton X-100 and 10% DMSO were added) for 1 or 2 days, under refrigeration. Excess fluid was removed from each slide and all slides were placed in an electrophoresis tank to which a basic solution (300 mM NaOH, 1 mM EDTA, pH > 13) was added. Slides were left to stand in this solution for 20 min to enable unwinding of DNA and expression of alkali-labile sites and single-strand breaks. Electrophoresis was then run for 20 min at 25 V, 300 mA, and 0.9 V/cm. Slides were removed from the electrophoresis tank, washed three times in neutralizing solution (0.4 M Tris, pH 7.5), rinsed three times with distilled water, and left to dry at room temperature. All procedures subsequent to blood collection were carried out in the absence of light. Slides were then fixed and silver-stained as described by Nadin *et al.* (2001). For assessment of DNA damage, 100 cells per sample were examined under light microscopy (x200 magnification). Cells were scored on a scale of 0 (no migration) to 4 (maximal migration) according to tail intensity (dimensions and shape) (Figure 2). Therefore, the total sum of damage scores for a sample of 100 cells (the damage index) ranged from 0 (no migration in any cell) to 400 (maximal migration in all cells). Patient and control slides were processed and analyzed together (Bagatini *et al.*, 2008; Maluf *et al.*, 2009).

### The cytokinesis-block micronucleus (CBMN) assay

**Figure 3:**
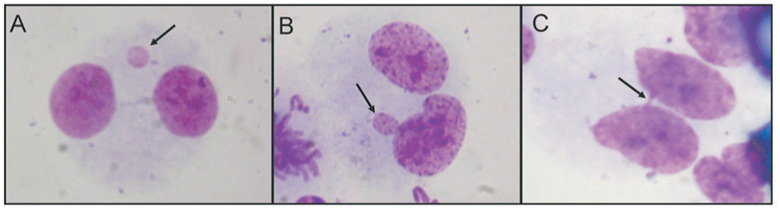
Nuclear abnormalities detected by micronuclei test (arrows). (A) micronucleus; (B) nuclear bud; (C) nucleoplasmic bridge.

For the CBMN assay, an aliquot of blood (0.5 mL) was added to 5 mL of RPMI 1640 medium supplemented with 20% fetal calf serum and 0.2% phytohemagglutinin. The culture flasks were incubated at 37 ºC for 44 h after which 4.5-μg/mL cytochalasin B was added. Twenty-eight hours later (72 hours of culture), the material was harvested according to the method described by Fenech and Morley (1985) and revised by Fenech and Crott (2002). The cell suspension was fixed in 3:1 methanol:acetic acid and dropped onto clean slides. The slides were then stained with Giemsa. One thousand binucleate cells from each individual were scored for micronuclei (MN), nucleoplasmic bridges (NB between daughter nuclei), and nuclear buds (BUD) (amplified DNA) (Figure 3) on slides identified by a code for blinded analysis.

### Statistical analysis

Results are presented as mean and standard deviation. Comparisons between groups were done using two-tailed Student's *t*-tests when the results followed a normal distribution, and the Mann-Whitney U-test was used when the results did not follow a normal distribution. Correlations between linear variables, such as the biochemical and DNA damage markers, were analyzed by Spearman's rank test. Statistical analysis was performed using SPSS 18.0, with a minimal level of significance of 0.05.

## Results

DNA damage levels detected by the comet assay were increased (*p*=0.023) in celiac patients (20.57 ± 10.59) when compared to controls (11.36 ± 6.42).

The frequency of micronuclei and nuclear buds were also increased in patients (6.66 ± 4.46 and 4.82 ± 3.90, respectively) when compared to controls (2.44 ± 2.14 and 2.60 ± 2.66, respectively) (*p* < 0.001 and *p*=0.007, respectively). There was no statistically significant difference between the frequency of nucleoplasmic bridges evaluated in patients and controls.

As for markers of oxidative stress, CAT and SOD activities showed significant differences between patients (10.17 ± 2.79 and 74.34 ± 22.23, respectively) and controls (6.48 ± 2.38 and 58.23 ± 23.29, respectively) (*p*=0.011 and *p*=0.013, respectively). However, GPx activity and lipoperoxidation levels measured as TBARS did not present statistic differences between celiac patients and controls.

The inflammatory markers MPO (938.21 ± 352.53) and NOx (54.32 ± 44.82), but not ADA, also showed increased values in patients in relation to controls (195.47 ± 39.84 and 31.15 ± 8.37, respectively) (*p* > 0.001 and *p*=0.009, respectively) (Table 1).

**Table 1:** Markers of oxidative stress, inflammation and DNA damage in patients with celiac disease and controls.

	Celiac disease	Controls	*p*
TBARS	0.060 ± 0.059 (n=47)	0.0433 ± 0.0432 (n=31)	NS
CAT	10.17 ± 2.79 (n=45)	6.48 ± 2.38 (n=20)	0.011
SOD	74.34 ± 22.23 (n=47)	58.23 ± 23.29 (n=20)	0.013
GPx	50.23 ± 8.98 (n=42)	53.13 ± 17.74 (n=20)	NS
MPO	938.32 ± 352.53 (n=47)	195.47 ± 39.84 (n=31)	< 0.001
ADA	13.06 ± 6.16 (n=46)	11.82 ± 2.14 (n=31)	NS
NOx	54.32 ± 44.82 (n=43)	31.15 ± 8.37 (n=31)	0.009
Comet	20.57 ± 10.59(n=44)	11.36 ± 6.42 (n=20)	0.023
MN	6.66 ± 4.46 (n=44)	2.44 ± 2.14 (n=25)	< 0.001
NPB	1.09 ± 1.18 (n=44)	0.92 ± 1.08 (n=25)	NS
BUD	4.82 ± 3.90 (n=44)	2.60 ± 2.66 (n=25)	0.007

Mean ± standard deviation. TBARS: Measure of substances that react with thiobarbituric acid; CAT: activity of catalase; SOD: superoxide dismutase activity; GPx: glutathione peroxidase activity; MPO: myeloperoxidase activity; ADA: adenosine deaminase; NOx: nitrogen oxides; Comet: Index of damage measured in 100 cells per individual; MN: micronucleus, NPB: nucleoplasmic bridge and BUD: nuclear buds, in 1000 cells per individual; P: significance level, NS: not statistically significant, n: number of subjects.

The relationship between different biomarkers was tested. DNA damage measured by the comet assay was correlated with CAT (r=0.405; *p*=0.009) and SOD activity (r=0.516; *p* < 0.001), and with the micronuclei frequency (r=0.77; *p* < 0.01). Positive correlations were also found between CAT and NOx (r= −0.315; *p*=0.042), GPx and NOx (r=0.349; *p*=0.030), and MPO and NOx (r=0.239; *p*=0.039).

## Discussion

When comparing patient and control groups, we found statistically significant differences in the contents of DNA damage assessed by the comet assay, as well as in the micronuclei and BUD frequencies that are genomic instability markers. Regarding the oxidative stress markers, CAT and SOD presented higher activity in CD patients, and as for the inflammation markers, MPO was lower and NOx was higher in patients compared to controls. The other markers analyzed in this study showed no statistically significant differences between patients and controls.

Pro-oxidative processes and inflammatory conditions are present in many diseases, including CD. It is well known that inflammation is associated with enhanced nitric oxide (^•^NO) levels, consequently leading to increased RNS and ROS generation (Ferretti *et al.*, 2012).

Accordingly, some studies suggest that gluten can generate oxidative stress (Szaflarska-Poplawska *et al.*, 2010; Ferretti *et al.*, 2012), although Szaflarska-Poplawska *et al.* (2010), analyzing urinary excretion of 8-oxodG and 8-oxo-Gua, have concluded that it may not be only the gluten that causes oxidative stress, and that there is a factor independent of diet. Cellular and tissue responses can be triggered by xenobiotics or endogenous compounds that act as a stimulus for inflammatory processes and oxidative stress. In CD, gluten is the agent that triggers the immune process that results in inflammation. Alfa-gliadin peptides enter the cell by endocytosis and accumulate in lysosomes, leading to the activation of signal transduction pathways and increasing the levels of ROS and RNS (Zimmer *et al.*, 2010).

Nitro-oxidative stress induced by gluten is involved in the activation of the transcription factor NF-κB that induces transcription of cytokines and enzymes, such as COX2 and iNOS, with consequent production of prostaglandins, ^•^NO metabolites, lipid hydroperoxides and other oxidative stress markers (Ferretti *et al.*, 2012).

As a consequence, CD patients usually reveal elevated ^•^NO levels both in serum and urine (Koster-Kamphuis *et al.*, 2003; Ertekin *et al.*, 2005), while evidence indicates that ^•^NO is also a regulator of intestinal inflammation (e.g., Kolios *et al.*, 2004). Therefore, CD is the result of a combination of severe nitro-oxidative stress associated with a chronic inflammatory process (Halliwell and Gutteridge, 2007). Accordingly, as mentioned before, in the present study we detected elevated NOx levels, as indirect measure of ^•^NO metabolites, which is an important participant in the inflammatory process (Lam *et al.*, 2015). The activity of MPO was enhanced approximately five times in CD patients, which is also responsible for exacerbation of the disease (Ferretti *et al.*, 2012). Interestingly, MPO activity as well as levels of TNF-α are higher in colitic rats compared to normal animals, while the treatment with an inflammatory plant extract was able to reduce such inflammatory markers (Abiodun and Oshinloye, 2017), revealing the importance of antioxidant supplementation in attenuating such condition.

In the present study SOD and CAT activity were up-regulated, nevertheless such increase apparently was not sufficient to prevent or even attenuate the systemic oxidative stress detected in CD patients. The positive correlations found between levels of DNA damage and SOD and CAT activities probably reflect the close interdependence of these two important antioxidant enzymes in counteracting the initial reactions of ROS generation, i.e., the formation of superoxide anion (O_2_^•-^) and hydrogen peroxide (H_2_O_2_), respectively. Therefore, they are able to attenuate the related nitrosative damage to important biomolecules and tissues promoted by the hydroxyl radical (^•^OH) generated by the Fenton/Haber-Weiss reactions (Wilhelm Filho *et al.*, 2000; Halliwell and Gutteridge, 2007).

Interestingly, and contrary to other studies, GPx activity was not altered compared to controls in the present study. A high GPx activity is generally found together with a high rate of peroxide production, as in erythrocytes of healthy subjects (Brigelius-Flohe, 1999). Taking into account that in the present study the TBARS contents found in plasma were not different in CD patients compared to controls, such maintenance of GPx activity could be justified. However, a positive correlation was found between GPx activity and NOx levels, despite relatively weak (r=0.349; *p*=0.030).

In a related study on the blood of CD patients, Stojiljkovic *et al.* (2007) found a marked increase in SOD activity, while GPx was significantly decreased. When measured in samples harvested from the small intestinal biopsies from CD children, SOD activity was also elevated, while the activities of GPx and GSR, as well as GSH levels were significantly decreased, thus showing an impairment of GSH-dependent enzymes (Stojiljkovic *et al.*, 2009). High levels of lipid oxidation (measured as TBARS levels) were found in the blood as well as in the small intestine mucosa of CD children in other related studies (Odetti *et al.*, 1998; Stojiljkovic *et al.*, 2009) respectively). All these results clearly indicate that the oxidative stress detected in the intestine mucosa is reflected in the blood of CD patients.

In addition to the consequences of ROS over-generation in CD as mentioned above, oxidative stress can also lead to higher rates of DNA damage. Misrepaired or nonrepaired DNA lesions result in mutations, which increase cancer risk. Chronic inflammation is strongly associated to human tumors, and one-fifth of all cancers have their formation route related to inflammation (De Marzo *et al.*, 2007).

Research in the area has shown that oxidative stress, chronic inflammation, DNA damage, and cancer are closely related conditions (Cooke *et al.*, 2006; Ferretti *et al.*, 2012; Ferguson *et al.*, 2015). Somatic mutations have been identified as causing neurological diseases (Poduri *et al.*, 2013) and other autoimmune diseases (Ross, 2014), in addition to cancer. Han *et al.* (2015), in a meta-analysis study, concluded that celiac patients have increased risk of all malignancies, including esophageal cancer and small intestine carcinoma, while Peters *et al.* (2003) showed an increased risk for non-Hodgkin's lymphoma and T cell adenocarcinoma of the small intestine in a Swedish cohort of celiac patients.

The first course of action after the diagnosis of celiac disease is establishing a gluten-free diet, which greatly improves the clinical symptoms of most patients (Pham-Short *et al.*, 2016). However, after this improvement in quality of life, patients begin to suffer from the specter of cross-contamination (See *et al.*, 2015), which is almost inevitable, taking into account the strong culture of wheat consumption of the western civilization. Celiac patients can have iron deficiency anemia (Rodrigo, 2006), because the absorption of iron is made in the proximal small intestine, which appears impaired in celiac disease. Szaflarska-Poplawska *et al.* (2010) postulated that control of oxidative stress and DNA damage found in the cells of celiac patients depends not only on gluten-free diet. They studied 8-oxodG levels and the mean plasma retinol and α-tocopherol concentrations in celiac patients and suggested a diet with vitamin supplementation to reduce cancer risk. CD is a condition caused by many factors. The undisputedly important environmental factor is gluten. The inflammatory process can lead to the development of cancer, but we have to consider that cancer is also influenced by several factors, and the individual's DNA repair capacity is one of the main factors. Therefore, patients with CD and DNA repair deficiency may be those most likely to develop cancer.

Therefore, taking into consideration the result of this study, it is highly suggested that patients supplement their diet with antioxidants and antimutagens, which can minimize the deleterious consequences of ROS over-generation associated with inflammation processes (Sram *et al.*, 2012; Ferguson *et al.*, 2015), eventually keeping the patient away from the threat of cancer onset or other diseases secondary to CD.

Another set of people who can benefit from dietary supplementation, are celiac relatives who carry the DQ2 and DQ8 genes and, therefore, may develop celiac disease. Avoiding oxidative stress, perhaps intestinal inflammation may not even start. As a consequence, supplementation with vitamins and minerals is highly recommended in such circumstances (Ferguson and Fenech, 2012).

The present study demonstrated that the inflammatory process and the systemic oxidative stress detected are closely related with DNA damage in CD patients. To avoid or attenuate such condition, removing the antigen (gluten), as well as counteracting ROS over-generation with an antioxidant intervention, might be key for CD treatment.
